# Statin Intolerance, Evolocumab for Hyperlipidemia, and Arrhythmia: Lessons Learnt From a Challenging Clinical Scenario

**DOI:** 10.7759/cureus.96864

**Published:** 2025-11-14

**Authors:** Matthew M Balocco, Rockib Uddin, Sara P Dimeo, Nezar Falluji, Abdul Waheed

**Affiliations:** 1 Emergency Medicine, Creighton University School of Medicine, Phoenix, USA; 2 Emergency Medicine Residency Program, Creighton University East Valley Arizona, Chandler, USA; 3 Division of Cardiology, Creighton University School of Medicine, Phoenix, USA; 4 Dignity Health Medical Group, Chandler Regional Medical Center, Chandler, USA; 5 Family and Community Medicine, Creighton University School of Medicine, Phoenix, USA; 6 Family Medicine, Dignity Health Medical Group, Gilbert, USA

**Keywords:** arrhythmia, atrial fibrillation, evolocumab, pcsk-9 inhibitors, statin-induced myositis, statin intolerance

## Abstract

PCSK9 inhibitors, such as evolocumab, provide a safe and effective therapeutic option for patients intolerant to statins. This is a report of a 78-year-old woman with extensive cardiovascular diseases including coronary artery disease, prior coronary artery bypass grafting, aortic and mitral valve replacements, stroke, hypertension, hypothyroidism, statin-induced myositis, and medication non-compliance, who required non-statin lipid therapy. After multiple statin failures due to side effects like myositis and discontinuation of ezetimibe for claimed gastrointestinal intolerance, she was prescribed a PCSK9 inhibitor. Four weeks later, she presented with elevated blood pressure and back pain. An electrocardiogram revealed atrial fibrillation (AF) with rapid ventricular response, which was a new development as the patient had no history of AF. Laboratory tests and chest imaging were unremarkable. She received intravenous diltiazem and was started on oral metoprolol and apixaban. Rate control proved challenging due to bradycardia, warranting the placement of a dual-chamber pacemaker. Evolocumab was resumed after discharge, and the patient remained clinically stable.

This case examines the occurrence of an arrhythmia, or AF, in a patient receiving evolocumab. It discusses contributing risk factors, underlying comorbidities, and clinical considerations, underscoring the value of reporting such events to expand understanding of potential, though uncommon, cardiac effects during PCSK9 inhibitor therapy. While no definitive association between evolocumab and AF was established, this case highlights the importance of monitoring for rare cardiac events in complex patients on PCSK9 inhibitors.

## Introduction

PCSK9 inhibitors, such as evolocumab, provide a potent and safe therapeutic option for patients with hyperlipidemia who cannot tolerate statins [1]. In a meta-analysis, evolocumab reduced LDL cholesterol by approximately 54% compared to placebo and PCSK9 inhibitors showed no significant increase in elevated liver enzymes, supporting their safety in patients with statin intolerance [1]. Additionally, these inhibitors have been shown to effectively lower cardiovascular event rates, addressing the challenges posed by statin-induced myositis in managing atherosclerotic cardiovascular disease (ASCVD) [2].

The safety profile of evolocumab has been well-documented in both clinical trials and real-world settings. Evolocumab has been proven to be generally safe for use, with most adverse effects (AEs) being mild and infrequent [3,4]. The most common AEs associated with evolocumab are injection-site reactions (such as pain, bruising, erythema, or hemorrhage) and muscle-related symptoms (especially myalgia and back pain), followed by less frequent events including arthralgia, muscle spasms, influenza-like illness, headache, non-specific pain, and new-onset diabetes [5]. Notably, a meta-analysis of 26 randomized controlled trials involving 62,337 patients at high cardiovascular risk showed no association between the use of evolocumab and increased risk of cardiac arrhythmia [6]. However, there has been one reported case of AF as an adverse reaction to evolocumab, which raises further questions about a possible causal relationship [7]. 

This is a case report of a patient with a prior history of statin-induced myositis and a high baseline cardiovascular risk who develops an arrhythmia shortly after evolocumab injection. This report aims to add to the literature regarding the safety of PCSK9 inhibitors and their association with AF.

## Case presentation

A 78-year-old woman with a complex cardiovascular history including hyperlipidemia, prediabetes, hypothyroidism, hypertension, statin-induced myositis, stroke, coronary artery disease (CAD), prior carotid endarterectomy, aortic and mitral valve replacements, and coronary artery bypass grafting (CABG), and medication non-compliance, presented to the clinic for follow-up. Data on the extent of her medication adherence are unavailable. She had previously failed multiple statins due to myalgias and elevated creatinine kinase (CK) levels and had discontinued ezetimibe for claimed gastrointestinal intolerance. Given her elevated ASCVD risk, she was started on PCSK9 inhibitor therapy with alirocumab. However, alirocumab was not on the patient's insurance formulary and was denied. The patient was then prescribed evolocumab at an equivalent dose, which was subsequently approved.

**Figure 1:**
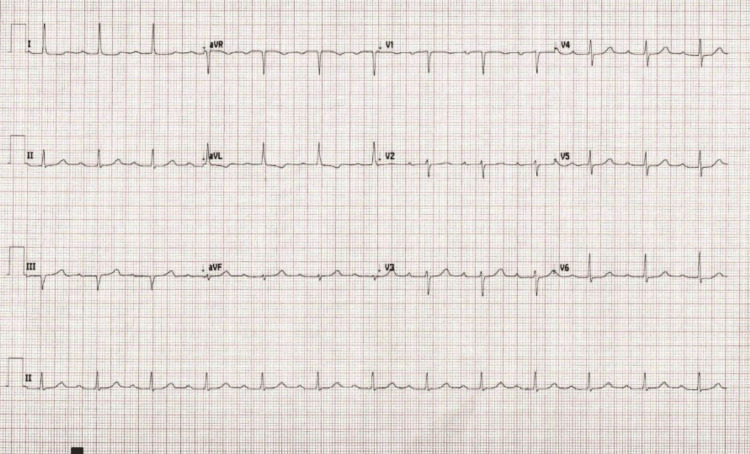
ECG taken several months prior to evolocumab administration.

**Figure 2:**
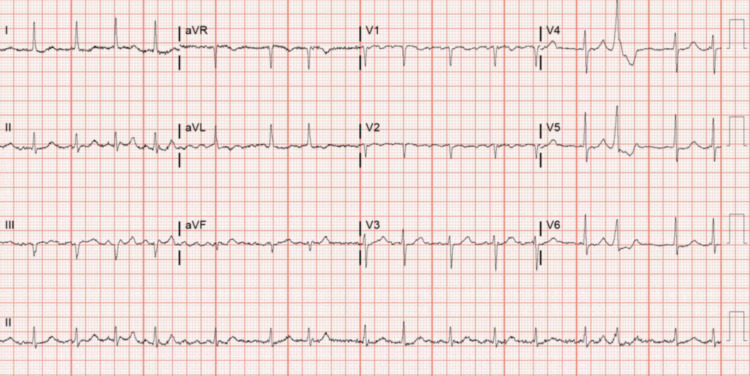
ECG taken upon arrival to the ED.

Four weeks after starting evolocumab, the patient presented to the clinic for back pain. She was noted to have an elevated blood pressure of 138/94 and a heart rate of 79. Other vitals were within normal limits. On physical exam, she was noted to have an irregular rhythm. An EKG was performed and revealed new-onset AF. Her most recent electrocardiogram (ECG) from the previous year had shown a normal sinus rhythm with a first-degree AV block, referenced in Figure 1. She was transferred to the emergency department and found to be in AF with a rapid ventricular response and heart rate of 101. Her blood pressure was 194/93, with other vitals unremarkable. Her EKG upon arrival is referenced in Figure 2. She had unremarkable laboratory findings, a normal chest X-ray, and a high-sensitivity troponin level of 5 ng/L. She received IV diltiazem due to its rapid onset and efficacy and was admitted for further management. Cardiology initiated oral metoprolol 25mg once daily. The patient's CHA₂DS₂-VASc score was calculated to be 6, so she was started on apixaban. During the first night of admission, telemetry revealed AF and adequate rate control, but there were several pauses. Her echocardiogram showed preserved ejection fraction with LVEF of 60-65% and a mildly dilated left atrium. Electrophysiology (EP) was consulted and recommended pacemaker placement because of concomitant tachy-brady syndrome.

**Figure 3:**
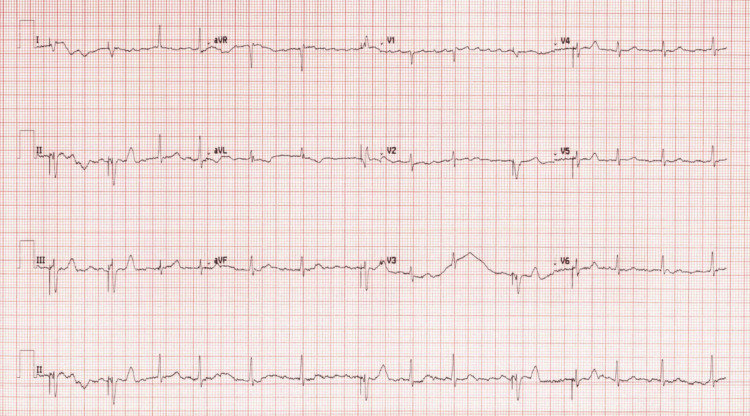
ECG at the three-month follow-up showing AF. AF: Atrial fibrillation

The patient underwent successful implantation of an Abbott dual-chamber pacemaker on hospital day four and was discharged the following day in stable condition after a brief observation period. Follow-up in both family medicine and EP clinics shortly after discharge confirmed she was doing well clinically and tolerating anticoagulation. Ezetimibe, which had been restarted in the hospital, was again discontinued due to elevated CK, and evolocumab therapy was resumed approximately one month after discharge. At her most recent follow-up in the EP clinic, two months after restarting evolocumab, she presented with AF and complained of intermittent episodes of palpitations. Her ECG showed AF with atrial-paced rhythm, referenced in Figure 3.

**Figure 4:**
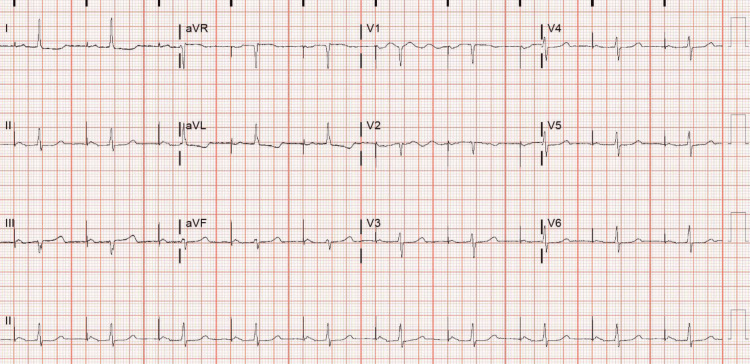
ECG showing an atrial-paced rhythm status post cardioversion.

The patient's device was interrogated and showed 100% AF burden with all parameters within normal limits. Two weeks later, the patient underwent transesophageal echocardiography and cardioversion. Her ECG after the procedure showed an atrial-paced rhythm, referenced in Figure 4. At her follow-up visit two weeks after the procedure, she remained in normal sinus rhythm and was stable on anticoagulation and evolocumab.

## Discussion

This case contributes to the growing literature on the safety profile of PCSK9 inhibitors, particularly evolocumab, in patients with high baseline cardiovascular risk. This patient had a significant ASCVD burden including CAD, prior CABG, valve replacements, stroke, hypertension, and prior statin intolerance due to myositis. She developed AF with a rapid ventricular response approximately four weeks after initiating evolocumab therapy.

There are limited documented cases of AF temporally associated with evolocumab; see Table 1 for a summary of the available case reports and comparison to the current patient.

**Table 1:** Comparison of a prior documented case report of atrial fibrillation associated with evolocumab, including age, sex, comorbidities, timing, and outcomes. Abdelmaseih et al. (2021) [7]. AF: Atrial fibrillation; CABG: coronary artery bypass grafting

^Case^	^Age (years)^	^Sex^	^Key Comorbidities^	^Timing of AF Onset^	^Outcome^
^Abdelmaseih et al. (2021) ^	^69^	^Female^	^Hypertension, hyperlipidemia, prior AF (s/p ablation two years prior)^	^Occurred after the second injection (~1 month)^	^Symptomatic AF with rapid ventricular response (HR 183 bpm); resolved with IV diltiazem; no recurrence after evolocumab discontinuation^
^Current Case Report^	^78^	^Female^	^Coronary artery disease, prior CABG/valve replacements, stroke, hypertension, hypothyroidism, statin-induced myositis, beta-blocker noncompliance^	^One month after initiation^	^New-onset AF with rapid ventricular response; treated with IV diltiazem, metoprolol, apixaban; pacemaker for tachy-brady syndrome; stable after resuming evolocumab^

Secondary analysis of the ODYSSEY OUTCOMES trial, a randomized controlled study of alirocumab in 18,924 patients with recent acute coronary syndrome published in the New England Journal of Medicine, showed very rare AF with no elevated arrhythmia risk [8]. A meta-analysis of 26 randomized controlled trials even suggested that PCSK9 inhibition might be associated with lower AF risk, contrasting with isolated case reports [6].

Postoperative AF is a well‑described complication after CABG surgery, with incidence ranging from 20% to 40%, which typically develops within the first week with a median time of two days after operation with most cases resolving and discharged in sinus rhythm [9]. Though this patient developed AF weeks after the surgery, the background risk remained elevated due to surgical stress, structural heart disease, and extensive atrial remodeling. 

This patient’s history of noncompliance and intermittent beta-blocker use may have also contributed to her new-onset AF. An analysis involving ~1,671 patients who had preoperative beta-blockers showed that if beta-blocker therapy was delayed beyond postoperative day 5 or omitted, postoperative AF occurred in 25% versus 16% in patients with early continuation, adjusted OR = 1.7 (95% CI 1.3-2.3; p < 0.001) [10]. Chronic beta-blocker use upregulates beta-adrenergic receptors, and abrupt cessation may trigger a sympathetic surge, causing tachycardia and increased myocardial stress, potentially contributing to this patient’s new-onset AF. However, confounding factors, including no recent surgery and uncertain noncompliance extent, limit definitive attribution.

In contrast, this case shares features with the previously reported case of evolocumab-associated AF, in which arrhythmia recurred after re-exposure, although the timing differs markedly. In this patient, AF reappeared only months after evolocumab was restarted, contrasting with the immediate recurrence described in the earlier report [7]. This delayed onset complicates assessment of a causal link and suggests that if a drug effect exists, it may not follow a predictable timeline. Clinicians should remain alert for arrhythmias in high-risk patients beginning PCSK9 inhibitor therapy, but such isolated observations should not preclude treatment when cardiovascular benefits outweigh potential risks.

## Conclusions

This case adds to the handful of reports suggesting a possible, albeit uncommon, association between evolocumab and AF. Although this patient experienced recurrence of AF after rechallenge, the delayed timing of onset makes a direct causal link to evolocumab questionable. Given her significant baseline risk, structural heart disease, and medication noncompliance, the temporal relationship between evolocumab and AF appears plausibly coincidental. Even so, this case highlights the importance of careful cardiovascular monitoring in patients with multiple risk factors who are initiated on PCSK9 inhibitors. Clinicians should remain vigilant for new arrhythmias, especially in older patients with structural heart disease or prior conduction abnormalities. While large trials have not demonstrated an increased risk of AF with PCSK9 inhibitors, individual case reports like this underscore the need for ongoing post-marketing surveillance. Ultimately, reporting such cases helps inform safe prescribing practices and guides risk-benefit discussions with patients requiring lipid-lowering therapy. Additional research and post-marketing surveillance are necessary to determine whether these reports reflect isolated events in particularly vulnerable individuals or indicate a true causal relationship.
